# GAS5 in Colorectal Cancer: A Multifaceted Long Non-Coding RNA in Tumor Suppression, Prognosis and Therapeutic Targeting

**DOI:** 10.34172/apb.025.45936

**Published:** 2025-12-21

**Authors:** Jiaxian Ong, Sharanya K Sritharan, Amar Harris Arifin, Baskaran Gunasekaran, Rhun Yian Koh, Nurulamin Abu Bakar, Shamala Salvamani

**Affiliations:** ^1^Division of Applied Biomedical Sciences and Biotechnology, School of Health Sciences, IMU University, Kuala Lumpur, Malaysia; ^2^Centre for Postgraduate Studies, IMU University, IMU University, Kuala Lumpur, Malaysia; ^3^Department of Biotechnology, Faculty of Applied Sciences, UCSI University, Kuala Lumpur, Malaysia; ^4^Centre for Diagnostics, Therapeutic and Investigative Studies (CODTIS), Faculty of Health Sciences, National University of Malaysia, Kuala Lumpur, Malaysia

**Keywords:** Colorectal cancer, Long non-coding RNA, GAS5, MicroRNA regulation, Competitive endogenous RNA, Biomarker

## Abstract

Colorectal cancer (CRC) is one of the most prevalent cancers worldwide, particularly affecting developed countries. Many pathways and mechanisms have been implicated in the development of CRC, and one of the targets of interest is the long non-coding ribonucleic acid (lncRNA), growth arrest specific 5 (GAS5). GAS5 has been observed to play an important role in many other cancers, and CRC is not an exception. This paper aims to understand the role and mechanisms played by GAS5 in relation to CRC progression. GAS5 has a complex role, with one of its major functions being its competitive endogenous nature that can downregulate multiple microRNAs such as miR-485-5p, miR-137, miR-26b, miR-21, miR-34a, miR-182-5p, miR-128-3p, and miR-221, which have implications in tumour growth and suppression. It is also observed to interact with proteins directly, such as the Yes-Associated Protein (YAP). Both clinical and laboratory evidence support the fact that in the context of CRC, GAS5 overexpression is a protective factor, while its underexpression is related to poorer prognosis and reduced survival. This has opened up its potential as a prognostic biomarker, as well as a therapeutic target to treat multiple cancers, including CRC. However, more research is still required to address existing knowledge gaps. In conclusion, GAS5 plays multiple roles and is an important player in cancer regulation, including CRC, and it holds good potential to be a biomarker and a therapeutic target.

## Introduction

 Colorectal cancer (CRC), the cancer of the colon and the rectum, is one of the most common cancers around the globe. According to the World Health Organization (WHO) Global Cancer Observatory (GLOBOCAN), it is the third most common cancer in men (10.6%) and the second most common in women (9.4%), just after breast cancer (24.5%).^1^ Overall, CRC ranks as the third most common cancer with an age-standardised rate (ASR) of 10.7 but is the second leading cause of cancer-related mortality with an ASR of 4.7. Notably, the more developed the country is, the higher the prevalence and risk of developing CRC. CRC develops through a multifactorial interplay of genomic instability, epigenetic changes, and environmental factors. About 70% of CRC cases result from chromosomal instability (CIN), which causes mutations in key genes such as APC, KRAS, TP53, and PI3KCA, subsequently disrupting cell cycle regulation and driving tumorigenesis.^2,3^ Other mechanisms include microsatellite instability (MSI), caused by defects in DNA mismatch repair, and the CpG island methylator phenotype (CIMP), which overlaps with MSI and involves extensive promoter hypermethylation and silencing of tumour suppressor genes.^2,3^ Together, these genetic and epigenetic alterations dysregulate critical signalling pathways, including Wnt/β-catenin, MAPK/ERK, and PI3K/AKT/mTOR, all of which drive tumour growth, progression, and therapy resistance.^2,3^ Additionally, environmental and lifestyle factors, such as diet, obesity, smoking, alcohol consumption, and chronic inflammation, contribute to CRC risk by aggravating DNA damage and influencing these molecular pathways. This highlights the complexity of CRC pathogenesis and underscores the need for targeted therapeutic strategies.^4^

 A consensus statement published by *Nature Reviews Molecular Cell Biology* defined long non-coding ribonucleic acid (lncRNA) as a class of non-coding RNA longer than 200 nucleotides.^5^ Similar to mRNAs, they undergo splicing, and although they can rarely have poly(oligonucleotide) tails, they are usually capped with 7-methylguanosine. They may also be derivatives of ‘pseudogenes’ and are generally less conserved than mRNA, with 3 major categories: intergenic, antisense, and intronic. A growing body of research has identified numerous lncRNAs associated with CRC, such as SNHG1, CRNDE, MIR17HG, H19, MAGI2-AS3, OR2A1-AS1, MALAT1, KCNQ1OT1, and the Growth Arrest Specific 5 (GAS5).^6–11^

 The GAS5 gene belongs to the 5’terminal oligo-pyrimidine class (5’TOP) and serves as a nucleolar RNA host gene containing 11 known C/D box small nucleolar RNAs (SNORD44, SNORD47, SNORD74-81, and SNORD103). Fifty (50) and 177 transcript/splice variants or isoforms of GAS5 have been reported and deposited in the GenBank and Ensembl databases, respectively, totalling 226 transcripts. These transcripts can prevent glucocorticoid receptor activation by direct DNA binding,^12–14^ and include an antisense region (GAS5-AS1). However, no study has yet validated or analysed these transcripts in the context of CRC. Further clinical and cell line research would therefore be beneficial, particularly using long-read sequencing technologies, as shotgun/ short-read sequencing can be susceptible to noise and artefacts.^15^

 The GAS5 lncRNA (Figure 1), also known as NCRNA00030 or SNHG2, is 846 nucleotides long and contains three functional modules: the 5’ module, the SR binding module, and the Core module. It is often found to be downregulated in many cancers, including CRC.^16^

**Figure 1:**
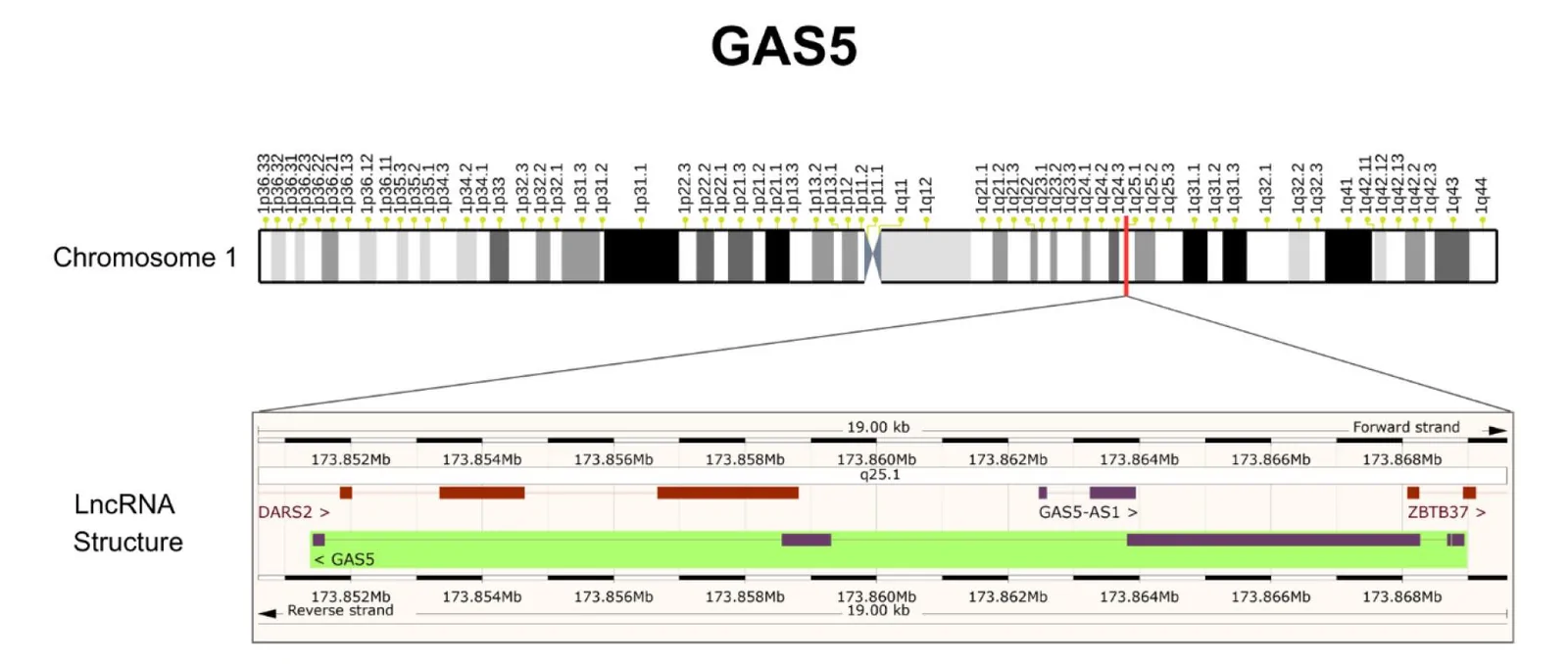
GAS5 structure from the Ensembl Genome Browser (ENSG00000234741), showing the entire region (highlighted in green). Located on chromosome 1 (q25.1): 173,851,424-173,868,940, transcribed from the reverse strand

 The RNA secondary structure of GAS5 remains poorly characterised and is thought to be complex and modular. Several studies have predicted its structure using bioinformatics tools such as SHAPE, RNA Structure, and the RNA Vienna Package.^16–18^ The only experimentally determined structure, obtained by X-ray diffraction, corresponds to the glucocorticoid response element (GRE) mimic, a conserved part of the SR module that influences GR-mediated transcription and is available under Protein Data Bank (PDB) ID number 4MCF (Figure 2).^19^

**Figure 2:**
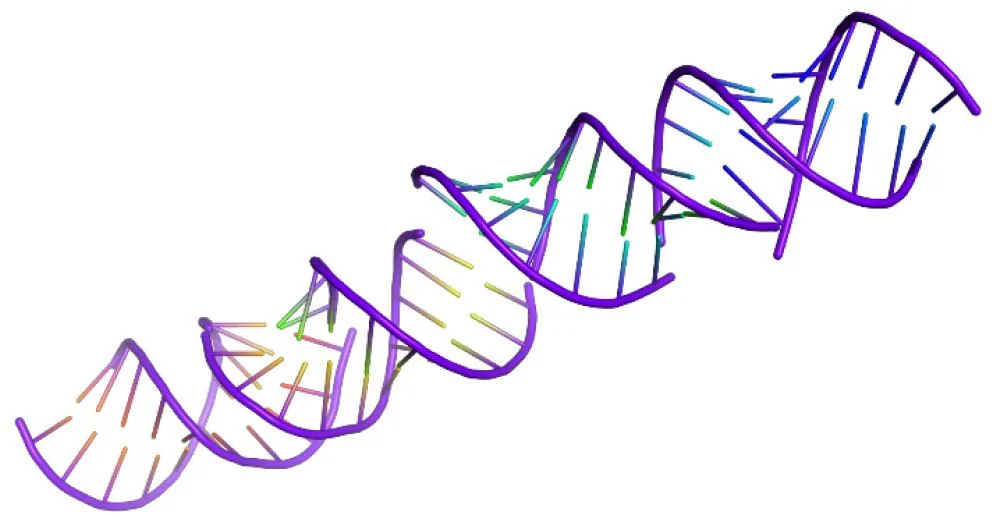
X-ray diffraction structure of the GAS5 glucocorticoid response element (GRE) mimic. PDB ID 4MCF, structure rendered using PyMOL 3.1.0.^19^

 GAS5 has become a gene of interest in cancer pathophysiology, biomarker, and precision medicine studies due to its promising potential. Previous reviews have discussed the roles and mechanisms of GAS5 in other cancers, such as prostate, breast, brain, pancreatic, ovarian, and cervical cancer, but none have addressed its role specifically in CRC.^20–27^ This review aims to highlight the current knowledge of lncRNA GAS5 and its significance in CRC. Furthermore, we discuss its oncogenic and tumour-suppressive mechanisms, molecular pathways and clinical potential as a biomarker and therapeutic target.

 To address the questions, this narrative review was compiled from a comprehensive literature search across multiple databases, including PubMed, Scopus, and Elicit, as well as publicly available datasets up to August 2025. Search terms included GAS5, LncRNA, Cancer/neoplasms, and CRC, with more specific terms to address relevant subtopics. Retractions and unreliable sources were excluded. The reference list of key papers was also examined for additional studies. The selected literature was compiled and synthesised thematically to discuss the mechanisms of GAS5 LncRNA, clinical implications, and emerging trends and challenges. Although this review is not systematic, great care was taken to include relevant and influential studies and to minimise selection bias.

## The significance of long non-coding RNA GAS5 in colorectal cancer

 Multiple studies have demonstrated that GAS5 can be detected in various locations in CRC patients, including tissue, blood plasma, and exosomes, indicating its diagnostic value.^28^ In fact, studies have shown that GAS5 is generally downregulated in CRC and that lower GAS5 levels are associated with a worse prognosis.^29^ For instance, Yin et al. first established that overexpression of GAS5 could inhibit cell proliferation both *in vitro* and *in vivo*.^30^ They also demonstrated that GAS5 was downregulated in human CRC tumour tissues, and this was correlated with tumour size, histological grade, and TNM stage.^30^ Therefore, their study concluded that GAS5 could be considered a potential prognostic factor in patients with CRC.^30^ Unfortunately, like many other studies on LncRNA GAS5, the paper has recently been retracted after 11 years of publishing, as the editors lost confidence in the results presented, as it was similar to other research and was missing key study details and methodologies.^31^ Since then, further follow-up studies have been conducted to understand the roles played by GAS5 and its related mechanisms, which will be discussed in the following section to look into this further.

 However, this finding was not entirely consistent. Wang et al. presented contradictory evidence, claiming that upregulation of GAS5 is associated with CRC. Specifically, they found that a mutation at the promoter region of GAS5 (SNP rs55829688 T > C) increases the binding affinity of Yin Yang-1 (YY1) to the GAS5 promoter, leading to an increase in GAS5 expression and subsequent promotion of CRC development. Thus, they suggested that this polymorphism is associated with an increased risk of CRC.^32^ Conversely, the rs145204276 polymorphism is another mutation noted at the GAS promoter region, but it was observed to reduce CRC risk and lymph metastasis in the Chinese population, but not in the Romanian population, suggesting possible population-specific genetic effects.^33^ A comparative summary of all the above clinical research can be found in Table 1.

**Table 1:** Comparative summary of clinical research data on GAS5 LncRNA and CRC risk. In most studies, downregulation of GAS5 is associated with a worse prognosis, with the exception of the rs55829688 T > C SNP variant. Other polymorphisms show more complex patterns, with rs145204276 indels demonstrating population-dependent and sometimes contradictory findings

**Research background**	**Sample size**	**GAS5 expression**	**GAS5 polymophism**	**Conclusion/ findings**	**Citation**
Jiangsu Province, China; in vitro validation	1078/1175	Upregulation	rs55829688 T > C	Increased CRC risk (OR > 1.50)	^32^
China (inferred Wuhan)	1400/1400	Not investigated	rs145204276 indels	Decreased CRC risk (OR = 0.79)	^33^
Romania (inferred Craiova)	156/195			Increased CRC risk (OR = 2.13)	^34^
Beijing, China; 7 cell lines	53/53	Downregulated	Not investigated	Increased CRC risk and advancement (staging, size, survivability, pathogenicity)	^35^
Nanjing, China; 4 cell lines	126				^36^
Xinjiang, China; SW480 cell line	158/173				^28^
Nanjing, China	66				^30^
Ningxia, China; xenographs	53				^37^
Jingzhou, China; 4 cell lines	24				^38^
Beijing, China; HT-29 cell line	24/24				^39^

 Aside from being a clinically significant lncRNA in colorectal cancer, reduced expression of GAS5 is linked to higher mortality as well. A recent paper published by Eisa et al. using the Tumour Immune Estimation Resource database found that GAS5 levels in colon adenocarcinoma are significantly higher than in normal cohorts.^40^ Higher GAS5 levels are seen in clinical cases to be protective, while low expression is associated with worse prognosis and overall survivability.,^35,36^ Kaplan-Meier charts of GAS5 expression in patients from Peking University People’s Hospital in Beijing, China (2010) revealed that, over 3 years, the survival rate was below 60%, compared to around 90% of patients with high GAS5 expression. A similar trend was reported in another study conducted at Huai’an First People’s Hospital, Nanjing Medical University, between 2011 and 2013. A Kaplan-Meier curve analysis was performed with pooled patient survival data from 4 different clinical studies, showing a significant correlation (P < 0.0001) between GAS5 LncRNA levels and mortality (Figure 3).

**Figure 3:**
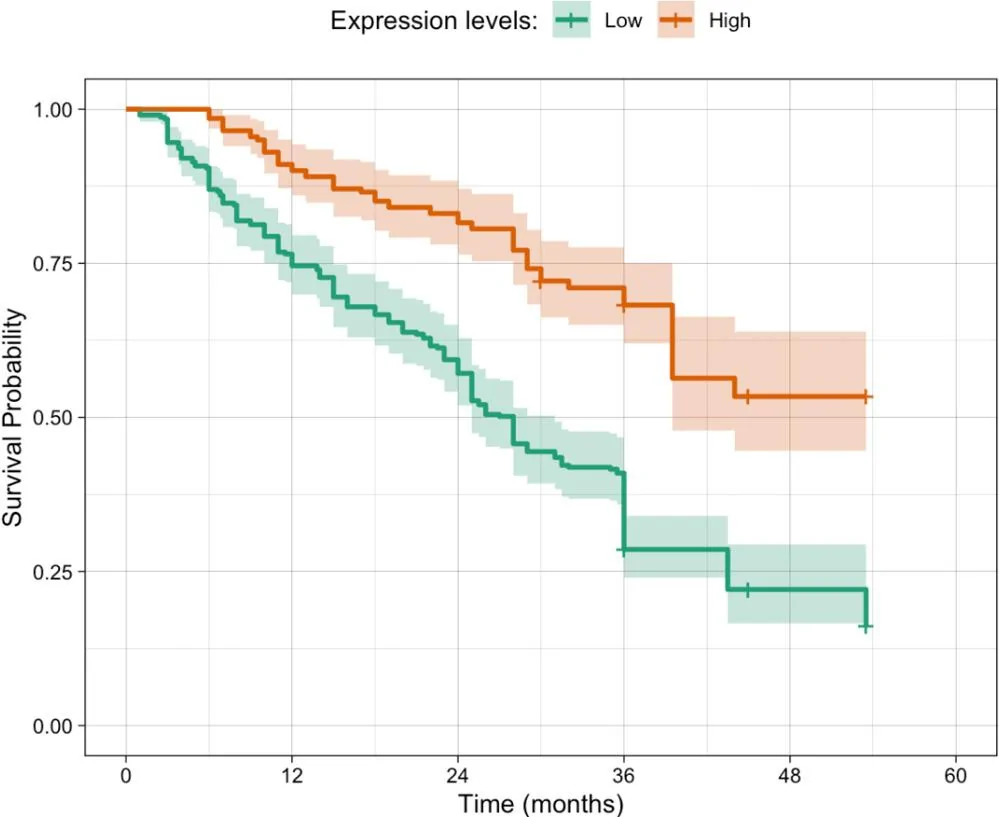
Pooled Kaplan-Meier estimator curve from 4 clinical studies showing correlation of LncRNA GAS5 tissue levels and patient survival.^28,35,36,39^ Reconstruction of the Individual Patient Data (IPD) from the clinical studies utilised IPDfromKM, where the survival fitting was performed using the survival R packages, respectively, for analysis.^41^ Total patients (n) = 516, *P* < 0.0001.

 Taken together, these observations suggest that GAS5 also holds prognostic significance for CRC. This is because reduced GAS5 expression has been correlated with advanced TNM stage, larger tumour size, and an increase in lymph node metastasis.^42^ Importantly, to advance GAS5 into clinical application, its prognostic role must be validated through robust, statistically powered patient cohorts. Moreover, studies evaluating hazard ratios, disease-free survival, and treatment response stratification based on GAS5 levels would further reinforce its utility. Notably, Liu et al. investigated the role of GAS5 in 158 CRC patients and reported that downregulation of GAS5 was associated with tumour size, tumour node metastasis (TNM) stages, Duke stages, lymph node metastasis (LNM), and recurrence rate.^28^ The fact that it is downregulated in CRC tissues compared to adjacent normal tissues indicates its potential as a biomarker for early detection and prognosis.^43^ Furthermore, GAS5 expression levels have been linked to overall survival rates, with patients exhibiting higher GAS5 expression generally showing better clinical outcomes.^39^ These findings highlight the importance of GAS5 in CRC pathogenesis and its potential as a therapeutic target in novel treatment strategies.

 To facilitate translation into clinical diagnostics, future efforts should prioritise assay standardisation across specimen types [e.g., plasma, exosomes, formalin-fixed paraffin-embedded (FFPE) tissue]. Validation of GAS5 expression using high-sensitivity techniques such as digital PCR or droplet-based assays and next-generation sequencing will be essential to ensure analytical reproducibility and clinical utility. Furthermore, multiplex biomarker panels that combine GAS5 with other CRC-associated lncRNAs or protein markers (e.g., CEA, miR-21, IL-2RG) may enhance diagnostic performance, particularly in early-stage or minimal residual disease detection.

 Moreover, CRC heterogeneity may influence the use of GAS5 as a biomarker. In fact, gene-expression-based frameworks, such as the Consensus Molecular Subtypes (CMS), capture the heterogeneity in CRC.^44^ The CMS classification categorises CRC into 4 subtypes, namely CMS1 (MSI Immune), CMS2 (Canonical), CMS3 (Metabolic), and CMS4 (Mesenchymal). Each subtype shows distinct molecular mechanisms, which could influence how GAS5 may function. For instance, in CMS1 tumours with active immune signatures, GAS5 might interact with immune-related pathways, while in CMS4 tumours, its role could be more closely tied to epithelial–mesenchymal transition (EMT) and invasion. Given this, GAS5’s prognostic significance may similarly vary by subtype, though this remains untested. Moreover, discrepancies in reported GAS5’s expression patterns in CRC may also reflect differences across CRC subtypes, although GAS5 has been widely reported to be tumour suppressive.

 These findings support GAS5’s prognostic and diagnostic potential, though future studies should incorporate subtype-specific analyses. From a diagnostic perspective, clinical translation requires evidence of sensitivity, specificity, or predictive value, which are critical for clinical relevance.

## Mechanism and roles of GAS5 in CRC

 GAS5 has emerged as an essential regulator in cancer progression, exhibiting multifaceted functions. In CRC, GAS5 is commonly downregulated and functions as a tumour suppressor by regulating apoptosis, proliferation, and migration pathways.^45^ A major mechanism involves its role as a competitive endogenous RNA (ceRNA), whereby GAS5 can sponge specific miRNAs to modulate the expression of downstream genes involved in tumour progression.^6,17^ Although molecular networks involving GAS5 have been identified, its full functional scope remains largely uncharacterised, with many mechanisms yet to be explored in the context of colorectal cancer. Given GAS5’s extensive ceRNA interactions and regulatory complexity, integration with transcriptomics, proteomics, and glycomics may help uncover additional downstream targets and regulatory feedback loops relevant to CRC pathophysiology.

 Supporting this, Lei et al. investigated the role of GAS5 in CRC using 48 clinical samples and the CCD-841, HCT116, SW620, LoVo, and HT29 cell lines.^38^ They demonstrated that GAS5 overexpression induces apoptosis through G1 phase arrest, and western blot analysis revealed that cyclin D1 was downregulated and p21 was upregulated, consistent with its tumour suppressive role. Importantly, these findings align with other reports showing GAS5 underexpression in CRC samples, where higher GAS5 expression appears protective, whereas lower expression correlates with an increased risk of metastasis.^16,17,28,33,35,38,39,42,46–52^

 When Lei et al. initially identified the involvement of Cyclin D1, the precise mechanism was unclear. More recent studies have since shown that GAS5 exerts its effects through ceRNA activity, sponging miRNAs that converge on key regulatory pathways, including FOXO, mTOR, PTEN/AKT, and NF-κB (miRNA unknown), all of which are known to regulate Cyclin D1 expression, apoptosis and proliferation.^16,38,39,48–50,53^ Notably, GAS5 has also been implicated in modulating the PI3K/AKT/mTOR pathway by influencing Wnt, AKT, FOXO, and mTOR, reinforcing its role in growth arrest and apoptosis.

 This review has identified ten molecular pathways associated with the pathogenesis of CRC based on GAS5 interactions, including eight known direct interactions with miRNAs, namely miR-221, miR-128-3p, miR-182-5p, miR-34a, miR-21, miR-26b, miR-137, and miR-485-5p, and a protein interaction with the Yes-associated protein (YAP) (Figure 4).^6,16,17,28,46-51,54^ Beyond these, GAS5 also influences pathways that affect cytokine secretions, angiogenesis, immune evasion, and ribosome biogenesis.^6,39,47^ Hence, the molecular mechanisms and roles of GAS5 in CRC are discussed in detail below.

**Figure 4:**
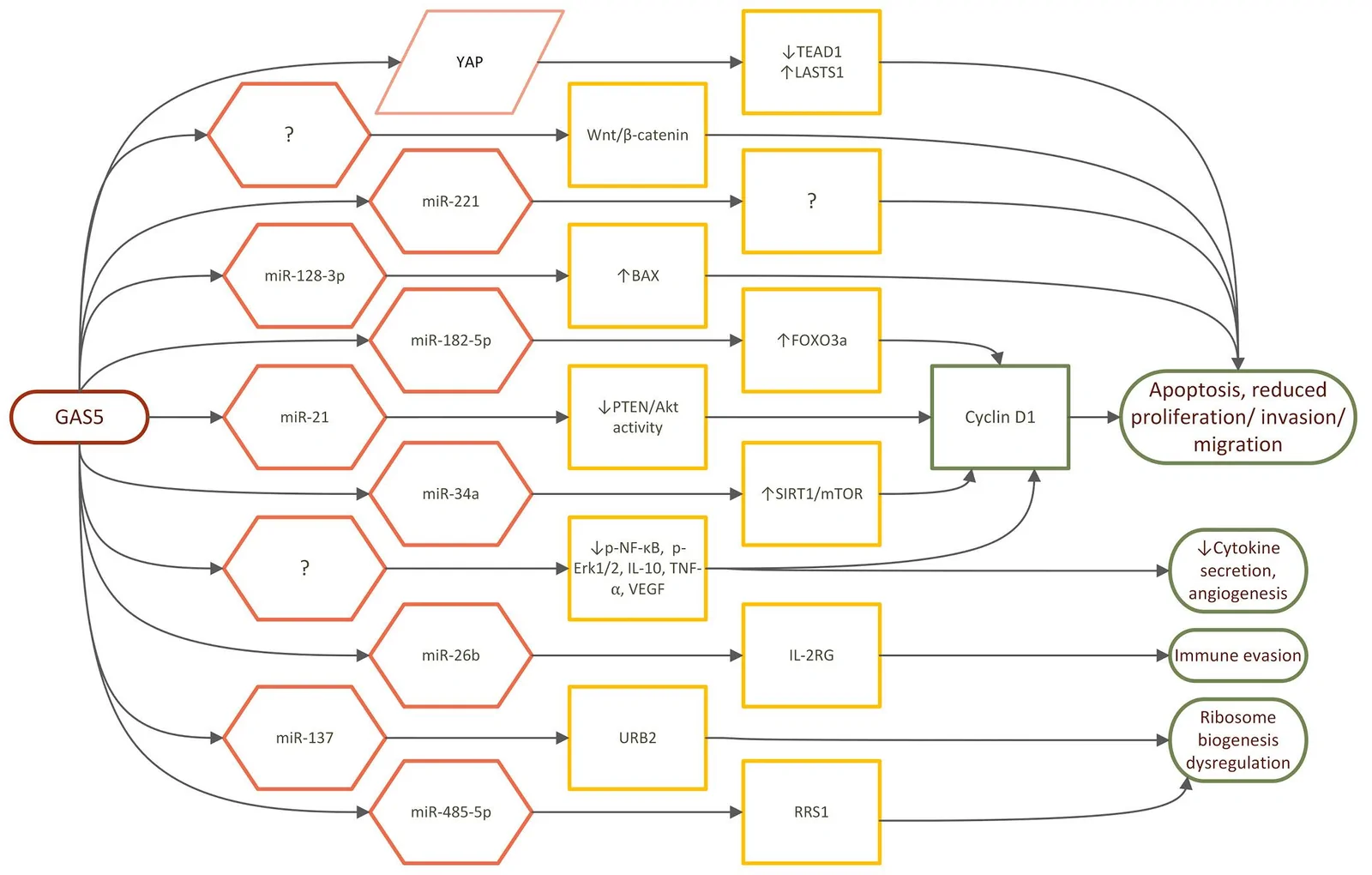
Summary of interactions of GAS5 and effects towards colorectal cells. Trapezoid: associated protein, hexagons: miRNAs (ceRNA target), rectangle: pathway/mechanisms involved, oval: outcome/effects, question marks (?): Unknown factors. Constructed using Microsoft Visio

###  Interactions between GAS5 and miRNA Networks Involved in CRC

####  miR-34a (SIRT1/mTOR/GAS5 axis)

 Krell et al. first identified the association of GAS5 and miR-34a in CRC, showing that this mechanism involved GAS5-derived small nucleolar RNA (snoRNA) and is independent of DICER, as it still functions in a DICER knockdown experiment.^46^ Furthermore, Zhang et al. demonstrated that GAS5 regulates macroautophagy and apoptosis in CRC cells via a negative feedback loop, partly explaining how CRC cell macroautophagy is in equilibrium with strong anti-apoptosis characteristics during progression.^50^ Mechanistically, GAS5 sponges miR-34a, leading to its reduced expression. In fact, miR-34a suppresses the Silent mating-type information regulation 2 homolog 1 protein (Sirtuin 1, SIRT1), which enables the activation of the p53 pathway. Therefore, GAS5 upregulation results in increased SIRT1 expression, inactivation of p53, and induction of apoptosis. Conversely, it is hypothesised that underexpression of GAS5 also triggers the miR-34a and SIRT1 axis, leading to phosphorylation and downregulation of the mammalian target of rapamycin (mTOR) pathway, ultimately resulting in the accumulation of GAS5.

 The studies used a wide variety of methodologies and validation approaches to confirm that GAS5 does play a role in CRC via the miR-34a/SIRT1/mTOR axis, including the use of clinical samples, mechanistic experiments, and *in vivo* validation. Although the clinical findings revealed a high variability in GAS5 levels among samples, the modest sample size and variance still showed a significant reduction in GAS5 levels, and did not undermine the central conclusions. However, further studies and validation would strengthen these findings.

####  miR-21 (PTEN/AKT) 

 Through an *in vitro* study on HCT-116, GAS5 was shown to act as a ceRNA against miR-21.^48^ Upon GAS5 silencing, western blot analysis showed increased Snail, N-cadherin, vimentin, Sox2, CD44, Oct2, alongside phosphorylated AKT, while E-cadherin and PTEN were decreased. It was therefore concluded that the reduction of GAS5 leads to the accumulation of miR-21, which activates the PTEN/AKT pathway, allowing for cancer survival in CRC patients. Validation was performed using luciferase, qRT-PCR, cell viability, and flow cytometry.

 Another similar study used clinically acquired samples to study the relationship of GAS5 and LIFR, and also found that miR-21 is being sponged by GAS5, upregulating LIFR.^37^ This suggests the involvement and interplay of LIFR and PTEN/AKT, which is supported by other past research done on other cancers.^55^ Further functional validation is still required to confirm the relationship of GAS5, miR-21, LIFR, PTEN, and AKT in the specific context of CRC.

####  miR-128-3p (BAX) 

 To investigate drug-resistant CRC, researchers in Korea identified that GAS5 regulates BAX expression in HCT116 cells.^54^ Mechanistically, GAS5 acted as a ceRNA against miR-128-3p, thereby restoring the expression of BAX, a pro-apoptotic protein. Consequently, BAX was overexpressed, promoting apoptosis. Moreover, bioinformatic analyses utilising BLAST revealed that BAX mRNA is capable of binding to GAS5 within the 3’UTR regions, specifically between 563-574 nt and 895-909 nt. Their study therefore showed that GAS5 competes with miR-128-3p, which typically suppresses BAX expression, thereby promoting apoptosis by restoring BAX activity.

####  miR-26b and IL-2RG 

 In a study by Gharib et al., bioinformatics analyses were used to identify GAS5 as a regulator of the interleukin-2 receptor gamma (IL-2RG). In fact, IL-2RG has potential in predicting CRC prognosis and could also be used as an immunotherapy target.^47^ They further discovered that GAS5 competes with has-miR-26b-5p, thereby enhancing IL-2RG expression. This interaction suggests that GAS5 may indirectly modulate immune evasion in CRC by increasing IL-2RG mRNA expression. The paper did not report additional validation of the network analysis, such as docking and binding simulations, but the authors acknowledged the need for experimental confirmation using luciferase reporter assays.

####  miR-182-5p FOXO3a pathway 

 Cheng et al. showed that GAS5 downregulation increased miR-182-5p levels, leading to decreased FOXO3a expression using western blotting from patient tissue samples and HCT-116, HT-29, SW480 and LoVo CRC cell lines.^49^ They concluded that the decreased GAS5 expression in CRC patients increases miR-182-5p levels, which in turn reduces FOXO3a expression, leading to enhanced proliferation and impaired apoptotic responses. The ceRNA effect of GAS5 on miR-182-5p was investigated and validated using bioinformatics (DIANA) and luciferase reporter assays, with flow cytometry and CCK-8 for viability and proliferation, respectively. This is consistent with GAS5’s characteristic role as a molecular sponge for miRNAs. However, the specific mechanism by which miR-182-5p suppresses FOXO3a has not yet been elucidated.

####  miR-137 and miR485-5p 

 Poursheikhani et al. performed an integrative bioinformatics analysis using differentially expressed gene (DEG) analysis on RNA-seq and miR-Seq data from clinical samples, followed by gene ontology (GO) and KEGG pathway identification with ceRNA network generation.^6^ From the network, they identified that GAS5 interacts with two distinct miRNAs, which are hsa-miR-485-5p, which regulates RRS1 and hsa-miR-137, which regulates URB2. However, the authors did not explain why GAS5 was included in the ceRNA network analysis, as GAS5 did not emerge from DEG and GO analyses, which may raise questions about the strength of evidence for its role in regulating colorectal cancer in this dataset. It is therefore important to validate these findings using wet lab techniques.

 Other potential limitations and challenges of this analysis lie in the reliance on databases with varied sources and quality, which would raise questions on batch variations, noise, and artefacts. The miRNA interaction evidence utilised by the tool, GDCRNATools, is based on databases as well, which could be incomplete, leading to missed targets. In addition, a strict significance threshold might have been used, which further explains the missing targets, notably by those elucidated by other tests via wet-lab investigations, which were discussed before. Conversely, it is also possible that some wet-lab investigations may not have captured all relevant interactions or may have introduced their own biases.

 However, the parameters used for the bioinformatic analysis and output data were not assessed due to code and record unavailability, and therefore, further comments could not be made. Further bioinformatics validation, such as molecular binding/docking simulations between the miRNAs and GAS5 to assess their affinity, could serve as an additional checkpoint and provide stronger supporting evidence. Regardless, wet-lab validation, such as luciferase assays, is still required to confirm these proposed interactions.

###  Interactions between GAS5 and proteins

####  Hippo YAP ubiquitination 

 GAS5 has been shown to interact directly with YAP, specifically through the WW domain at 171-263 aa with 262-480 nt.^17^ This interaction promotes YAP translocation from the nucleus to the cytoplasm, where it undergoes phosphorylation, notably at serine 127, followed by ubiquitin-mediated degradation. The overexpression of GAS5 suppresses YAP interaction with TEA domain transcription factor 1 (TEAD1) but facilitates interaction with 14-3-3 or LASTS1, and the opposite is observed when GAS5 is underexpressed.

 The authors also found that the knockdown of YTHDF3 (YTH domain-containing family protein 3) reduced CRC proliferation and metastasis and that YAP modulates YTHDF3 transcription by binding to the promoters. There seems to be a negative feedback loop in play as well, as YTHDF3 can promote the decay of GAS5, specifically those with m^6^A (N6-methyladenosine) modifications, by direct binding. In other words, RNA m^6^A modifications mark GAS5 transcripts, while YTHDF3 is a protein which contains the m^6^A reader domain YTH that helps recognise m^6^A-modified GAS5 for degradation, highlighting the role of epitranscriptome regulation of GAS5. It is later understood that YTHDF1 regulates the expression of TEAD1, but more research is needed to understand the exact mechanisms.^52^ Therefore, the overexpression of GAS5 is protective against CRC as it reduces YAP, suppressing interactions with TEAD1, enhances LASTS1 interactions, while GAS5 expression is regulated through epitranscriptome mechanisms and degraded by YTHDF3.

####  Wnt/β-catenin 

 Song et al. found that GAS5 overexpression suppresses cell invasion, especially in the SW480 CRC cell line. Through RT-qPCR and Western blotting analysis of LoVo, HCT116, SW480, and SW620 cell lines, it was established that the activation of the Wnt/β-catenin pathway contributed to the invasion.^42^ However, the exact mechanism by which GAS5 regulates the Wnt/β-catenin pathway remains unclear, and further research is required.

####  IL-10 and VEGF inhibition signal transduction protein

 An *in vitro* study using DLD-1, HCT-116, HT-29, SW620, and SW480 cells found that GAS5 affects cytokine secretion by CRC cells, notably VEGF-A and IL-10.^39^ In CRC, the reduction of GAS5 led to increased levels of phosphorylated NF-κB, phosphorylated Erk1/2, IL-10, TNF-⍺, and VEGF-A, allowing CRC cells to have a higher proliferation rate as well as colony-forming ability. VEGF is involved in angiogenesis, which explains how CRC can grow so effectively by forming additional blood vessels for nutrients.

## Unexplored mechanisms

 Aside from the previously discussed mechanisms and roles played by GAS5 LncRNA, there are other pathways investigated in other cancers but not yet specifically explored in CRC. Several reviews have extensively examined GAS5 in different malignancies, such as breast, glioma, ovarian, prostate, and gastric cancer, or as part of general lncRNA studies.^21,56–63^

 Among these, breast cancer models are the most thoroughly investigated, with the mTOR pathways being the most studied pathway due to their critical role in apoptosis and responsiveness to Rapamycin. Studies have also extended this mechanism to leukaemic T-cells.^64^ Another interesting but less explored pathway is SUFU signalling, where GAS5 binds directly to miRNA-3781-5p, which plays a role in SUFU regulation, and its increase could make them more sensitive to paclitaxel.^65^ In cervical cancer, miR-106b was identified as a target which plays a role in regulating the immediate early response 3 gene (IER3).^66^ It is also noted that this reciprocal function of GAS5 towards the mTOR pathway is also found in androgen-dependent prostate cancer cells, where the administration of mTOR inhibitors resulted in higher GAS5 expression in a different study.

 In ovarian cancer, miR-23a was found to mediate malignant phenotypes through the WT1 axis.^67^ The AKT signalling is also affected through the hnRNPK axis.^68^ Epigenetically, SNORD75, derived from GAS5 intron, participates in the production of the Pi-sno-75, which interacts with the Piwi proteins, PIWIL1 and 4. This complex contributes to histone modifications in TRAIL in breast cancer through the recruitment of MML/COMPASS-like complexes, forming the piRNA/MLL3/UTX regulatory assembly.^69^

 With respect to FOXO signalling, studies in glioma, breast and cervical cancer revealed that GAS5 suppresses miR-196a-5p and miR-205, thereby modulating the FOXO1-PI3K/AKT pathway.^70–72^ This pathway also plays a role in regulating GAS5 transcription through promoter binding, leading to MIIP and PID1 expression and establishing a positive feedback loop. Therefore, these mechanisms should be further investigated and validated in CRC models to confirm their relevance.

 In summary, these mechanisms remain unverified in CRC but may offer valuable insights and future directions for mechanistic and translational research.

## Current therapeutics targeting GAS5

 Targeting GAS5 has been proposed as a promising strategy for the development of novel therapeutics in colorectal cancer. RNA-based therapeutic modalities such as GAS5 mimics, antisense oligonucleotides (ASOs), or CRISPR-based transcriptional activators (e.g., CRISPRa) could represent innovative and precision-driven approaches to restore GAS5 function in resistant or advanced CRC cases. By modulating GAS5, these strategies may overcome pathway redundancies or feedback loops that reduce the efficacy of conventional monotherapies. The multitarget effects of GAS5, which include regulation of apoptosis, epithelial–mesenchymal transition (EMT), immune modulation and drug sensitivity, also suggest a lower risk of resistance development, making it an attractive therapeutic target. This section discusses the potential of GAS5 as a therapeutic target, focusing on current strategies under investigation, including gene therapy, anti-cancer drugs and natural compounds that may modulate its expression or function.

###  Gene Therapy 

 One of the strategies to restore GAS5 activity in CRC involves the use of oncolytic adenoviruses. Yuan et al. developed an SPDD-modified oncolytic adenovirus (survivin promoter‐E1A 24bp deletion‐E1B deletion) engineered to coexpress GAS5 and snoRNA p44.^53^ In CRC cell lines SW620 and LS174T, viral-mediated GAS5 delivery significantly inhibited cell proliferation and induced cell apoptosis.^53^ Moreover, intratumoral injection of the adenovirus significantly suppressed xenograft tumour growth in nude mice.^53^ When combined with Rapamycin, an mTOR inhibitor, a greater suppressive effect was observed. These findings suggest that GAS5 restoration could synergise with pathway-targeted therapies to suppress CRC progression.^53^

 Beyond viral delivery, nucleic acid–based interventions such as antisense oligonucleotides (ASOs) and CRISPR-based transcriptional activators (CRISPRa) may also be considered. Although ASOs directly targeting GAS5 have not yet been tested in CRC, ASO-based silencing of oncogenic lncRNAs such as HOTAIR has shown efficacy in CRC.^73^ Liu et al. demonstrated that knocking down HOTAIR in CRC cells using siRNA or shRNA decreased cell viability and reduced autophagy in both *in vitro* and xenograft models.^73^ Given that GAS5 is consistently downregulated in CRC, synthetic ASO mimics designed to stabilise GAS5 transcripts or prevent degradation could be explored as a therapeutic approach. On the other hand, CRISPRa technology which employs nuclease-deficient Cas9 fused to transcriptional activators, has already been applied in CRC research to investigate lncRNA function.^74,75^ Therefore, this approach could be adapted to selectively enhance endogenous GAS5 expression and restore its tumour-suppressive activity. Although no GAS5-specific CRISPRa studies have yet been published to date, the feasibility of this approach offers a promising direction for future research.

###  Anti-cancer drugs 

 In the only study directly linking GAS5 to chemotherapy resistance in CRC, Kim et al. demonstrated that upregulating GAS5 expression in HCT116 cell lines increased the chemosensitivity to 5-fluorouracil (5-FU). Consequently, BAX was overexpressed, promoting apoptosis.^54^ Therefore, their study highlighted GAS5 as a potential chemosensitiser for patients with 5-FU–resistant CRC, which remains a major clinical challenge. Such evidence suggests that therapeutic delivery of GAS5 could be integrated into standard chemotherapy regimens to improve efficacy and delay resistance.

 Additional evidence also points to a broader role for GAS5 in modulating drug response. Interestingly, verteporfin, originally thought to inhibit YAP1, was later shown not to act on the protein itself but rather on the impaired clearance of protein oligomers in CRC.^76^ Although not directly linked to GAS5, verteporfin underscores how drugs with unexpected molecular targets may intersect with lncRNA-regulated signalling pathways, suggesting that GAS5-associated networks could be indirectly modulated by existing pharmacologic agents.

 Insights from other malignancies further strengthen the rationale for exploring GAS5 in CRC drug resistance. For instance, increasing GAS5 expression has been shown to increase the sensitivity of cervical cancer cells to cisplatin.^77^ Moreover, ailanthone administration enhanced GAS5 expression by targeting the UPF1/GAS5/ULK1 pathway, where it suppressed mRNA degradation through nonsense-mediated decay mediated by UPF1 in non-small lung cancer.^78^ While these observations were derived from non-CRC models, the shared signalling pathways suggest potential translational parallels in colorectal tumours. Applying such findings in drug-resistant CRC models could identify combination strategies leveraging GAS5’s tumour-suppressive effects. A preclinical study could involve testing a CRC cell line resistant to standard chemotherapeutics, followed by genetic or pharmacological manipulation of GAS5 expression. Subsequent evaluation of cell viability, apoptosis induction, and downstream signalling pathways would clarify whether combining GAS5 modulation with chemotherapy enhances treatment efficacy and mitigates resistance mechanisms in CRC.

###  Natural products

 The evidence for natural products that modulate GAS5 expression in CRC is still lacking, but findings from other cancers and preliminary studies provide important leads that warrant further investigation. One such compound is 2-O-methylmagnolol (MM1), a magnolol derivative extracted from the bark of the *Magnolia officinalis* plant.^79^ MM1 was found to upregulate GAS5 and induce apoptosis in keratinocytes, with greater potency than magnolol itself. However, these results have not been validated in CRC cell lines and given the established role of GAS5 in promoting apoptosis and inhibiting proliferation in CRC, magnolol derivatives may hold therapeutic relevance and could be investigated further.

 Another compound of interest is gambogic acid, derived from the resin of *Garcinia* species, more commonly known as a sap tree or mangosteen tree.^80^ In bladder cancer cells, gambogic acid was reported to increase GAS5 expression while simultaneously suppressing EZH2, a histone methyltransferase known to act as an oncogenic driver. EZH2 is frequently overexpressed in CRC, where it is associated with enhanced proliferation and poorer patient outcomes.^81^ This suggests a testable hypothesis that gambogic acid may demonstrate similar effects in CRC, especially in molecular subtypes characterised by high EZH2 activity.

 It would be scientifically valuable to extend these findings into CRC models, including chemo-resistant cell lines and patient-derived organoids, to assess GAS5 modulation by natural products in a more clinically relevant setting. Mechanistic studies should also evaluate whether such compounds influence GAS5 expression directly or via upstream regulators, such as epigenetic modifiers, RNA-binding proteins, or miRNA networks.

## Limitations, future prospects and suggestions

 Overall, research on GAS5 in CRC has slowed down over the years, with multiple retractions due to data fabrication, resulting in a loss of trust in some lncRNA-related findings. From the research done to investigate GAS5’s interactions through wet lab studies, it is evident that this work is highly resource-intensive in terms of cost, technical skills and time. The research usually requires expertise in gene silencing, RT-qPCR, western blotting, and luciferase assays, to name a few, and demands rigorous experimental design to ensure the conclusion is sound and to minimise uncertainty. This leads to the need to purchase numerous expensive reagents, invest considerable time in training personnel, and allocate long experimental timelines. The bioinformatics analysis is also challenging and relies on a variety of specialised skills.

 Considering there are many unknowns regarding the mechanism of GAS5 interactions, substantial fundamental research is still needed to determine the unknown factors. There is also limited ongoing research on GAS5’s role in CRC and its therapeutic potential. Although studies on GAS5 exist in other cancer types, these findings must be validated and translated carefully to the CRC context. Besides, the antisense of GAS5 lncRNA (GAS5-AS1) has never been investigated before in the context of CRC. Past research has looked into GAS5-AS1 in cervical cancer, hepatocellular carcinoma, paediatric irritable bowel disease, and non-small cell lung cancer, and all concluded that GAS5-AS1 improves GAS5 lncRNA’s stability and that its downregulation will reduce GAS5 lncRNA as well, increasing cancer risk.^82–85^ Therefore, we hypothesise that a similar conclusion could be observed in the context of CRC, where GAS5-AS1 would improve GAS5 LncRNA stability, which would in turn increase GAS5 expression, reducing CRC risk. It would also be reasonable to assume that mutations in the GAS5-AS1 region would have the opposite effect, increasing CRC risk, which is also shown in cervical cancer.^82^ Future studies could look into this to determine the relationship between CRC and GAS5-AS1 through co-expression analysis, long-read sequencing technologies, and functional assays.

 The methodologies used in the investigation of GAS5 should also be critically evaluated, with many studies utilising silencing and pull-down experiments, with detection through western blotting. It is worth exploring emerging techniques and technologies to overcome technical shortcomings. For example, it should be more accurate to perform knockout experiments using CRISPR interference (CRISPRi) or CRISPR activation (CRISPRa). Moreover, a comprehensive multi-omics workflow for GAS5 could be designed as follows, where future researchers could begin with transcriptomics using RNA sequencing (RNA-seq) and single-cell RNA-seq (scRNA-seq) to profile GAS5 expression and its co-expressed transcripts across heterogeneous CRC tumour populations or even cell lines. Concurrently, Chromatin Isolation by RNA Purification followed by sequencing (ChIRP-seq) can be utilised to map the genomic binding sites of GAS5, revealing its interaction with chromatin and regulatory DNA elements.^86^ Proteomic analysis via RNA immunoprecipitation sequencing (RIP-seq) or cross-linking immunoprecipitation (CLIP-seq) coupled with mass spectrometry would identify proteins directly interacting with GAS5, building a comprehensive RNA-protein interactome. Integration of epigenomics data, such as histone modification, ChIRP-seq and DNA methylation profiles, will enable correlation between GAS5 binding and epigenetic regulation relevant to CRC pathogenesis.

 By combining these techniques, the multi-omics approach aims to highlight the multidimensional regulatory network of GAS5, including competing endogenous RNA (ceRNA) interactions and epigenetic modulation of downstream targets like PTEN/AKT/mTOR and Wnt/β-catenin pathways. Expected outcomes include identifying novel GAS5 interactors, mechanistic insights into its tumour suppressive function, and potential therapeutic targets. This workflow would enhance understanding of GAS5 at multiple regulatory layers in CRC, directly supporting translational biomarker and drug discovery efforts.^6^

 In addition, current methodologies studying GAS5, such as gene silencing via siRNA or shRNA and CRISPR-based knockouts, while proven to be useful, also come with limitations. RNA interference (RNAi) approaches may lead to incomplete knockdown and off-target effects, whereas CRISPR-Cas9 genome editing can introduce permanent DNA breaks, potentially triggering compensatory cellular responses and affecting neighbouring genes.^5^ Additionally, bulk RNA assays mask cellular heterogeneity, making it challenging to capture the full spectrum of the role of GAS5 in diverse CRC cell populations or tumour microenvironment cells.

 To overcome these challenges, advanced techniques such as single-cell RNA sequencing (scRNA-seq) allow high-resolution mapping of GAS5 expression dynamics at the single-cell level, differentiating cell type-specific functions and providing information on intratumoral heterogeneity.^5^ Cryo-electron microscopy (Cryo-EM) can provide near-atomic structural insights into GAS5-RNA-protein complexes, revealing mechanistic details of its folding and interactions with spliceosomal and RNA-binding proteins under physiological conditions.^42^ Integration of these technologies offers improved precision and mechanistic understanding that are essential for developing targeted therapies and for functional validation of GAS5’s role in CRC progression.^5^

 Other than that, the review identified multiple isoforms of GAS5, which were not annotated and characterised well, with potentially many variants missed during sequencing. Researchers should consider using long-read technology, such as PacBio and Oxford Nanopore (ONT) sequencing to identify them.^87,88^ Besides that, there are no confirmatory studies to identify the structures of GAS5 experimentally, such as CryoEM and Illumina SHAPE-MaP.^89^ Future work should therefore prioritise systematic isoform annotation and structural characterisation. It is important to remember that correlation should be interpreted carefully, as the results may correlate, but this might not be the actual causation or mechanism. The review also outlined that there are many targets of GAS5, which might result in off-target cross-reactions from the study design, as well as off-target result collection due to the wide range of targets. This would mean that more validation studies must be done to address all possible explanations or loopholes, such as more mutation mapping and co-immunoprecipitation experiments.

 Since it has many targets, it might be worthwhile to investigate the targets of GAS5 through multi-omics strategies such as whole transcriptome and proteome sequencing and profiling. This allows a full picture of the targets and intermediaries of GAS5, as well as the associated protein expression and pathways affected. Transcriptome analysis could not only provide all significant differentially expressed transcripts, but further analysis could be done to identify KEGG and Gene Ontology, as well as transcript splicing studies, variant calling, and, with spatial transcriptome, the location of the transcripts. A comprehensive picture of all the methodologies and technological advancements mentioned above is outlined in Figure 5.

**Figure 5:**
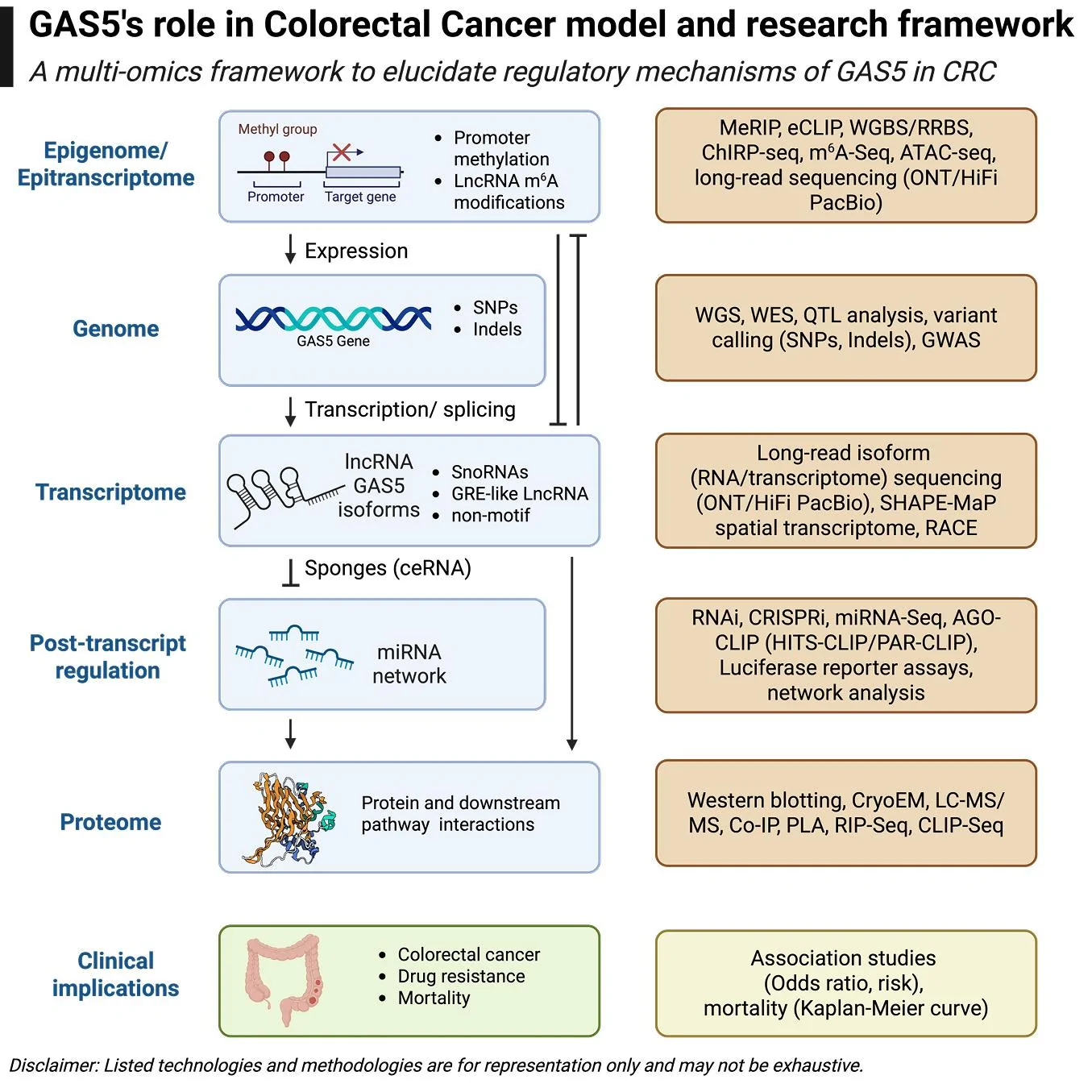
Model of GAS5 LncRNA’s role in colorectal cancer and a multi-omics research framework for the investigations of its various mechanisms and clinical implications. Disclaimer: Listed technologies and methodologies are for representation only and may not be exhaustive

 However, although the multi-omics approach may give a full picture of GAS5 targets and their intermediaries, it is susceptible to analysis biases. Further research would be required to look into specific mechanisms to understand how the transcriptome and proteome change from GAS5 isoforms, mutations, and expression levels. While GAS5 has shown promise as a diagnostic and prognostic biomarker in CRC, a more detailed evaluation using diagnostic metrics such as Receiver Operating Characteristic (ROC) curves, Area Under the Curve (AUC), sensitivity, specificity, and predictive values is essential to validate any potential clinical utility.^37^ Incorporating these statistical measures in future studies would greatly strengthen the quantification of GAS5’s ability to discriminate CRC patients from healthy individuals or to predict treatment response and disease progression. Practical considerations, including sample type like tissue, blood and exosomes, stability of GAS5 RNA, and standardisation of detection methods such as RT-qPCR, should be systematically examined to ensure reproducible clinical translation.^37^

 Regarding therapeutic targeting, the delivery of RNA-based therapies remains a major challenge due to instability, off-target effects, and inefficient uptake by cancer cells. Strategies such as nanoparticle-mediated delivery, lipid-based carriers, and tumour-specific ligands are promising for enhancing the GAS5 lncRNA mimics or ASOs bioavailability and specificity.^5^ Moreover, combination therapies integrating GAS5 modulation with chemotherapy or immunotherapy should be explored to overcome drug resistance seen in CRC.

 In the recent pan-cancer GAS5 bioinformatics analysis paper, differentially expressed genes (DEGs), mRNA co-expression network, KEGG, and for the ceRNA properties, CIRCOS and heatmap analysis and data visualisation were done using the Gene Expression Omnibus (GEO), Long Noncoding RNA Cancer Arrays (lnCAR), and Gene Expression Profiling Interactive Analysis (GEPIA) databases.^40^ This is intriguing and gives a comprehensive idea of potential GAS5 targets and their effects, and it could be expanded to the context of colorectal cancer. Undeniably, more data still needs to be collected for analysis for CRC.

 Many bioinformatics research papers require more validation through wet lab techniques as well as with patient samples, and more bioinformatics research is required to identify more links and leads on GAS5 interactions and drug discovery. There is little research on drugs targeting GAS5, but many more have not yet been tested on CRC, but on other cancers as well, and more natural product research should also look into the effects towards GAS5. Lastly, on its biomarker potential, translational and clinical research should be carried out to investigate the prevalence of GAS5 in actual patients and the feasibility of being deployed in a clinical setting.

## Future research roadmap

 Despite significant advances in understanding the tumour-suppressive role of GAS5 in CRC, several critical avenues are yet to be explored to fully understand its clinical potential. The following research roadmap outlines prioritised molecular targets and recommended study designs to guide future research.

 Firstly, the molecular target is YAP. As a key effector in the Hippo signalling pathway, YAP has been implicated in CRC progression and therapy resistance.^17^ Elucidating the mechanistic interplay between GAS5 and YAP, including direct or indirect regulatory effects, should be prioritised. Functional studies using CRISPR/Cas9-mediated gene editing and RNA immunoprecipitation can clarify these interactions.

 Besides, miR-34a is a well-established tumour suppressor microRNA, and evidence suggests that GAS5 may modulate its expression or activity since GAS5 itself is a tumour suppressor gene.^50^ Future research should focus on dissecting the GAS5/miR-34a axis using *in vitro* and *in vivo* CRC models, with particular attention to downstream effects on cell proliferation, apoptosis, and metastasis.

 Next, following the molecular targets of YAP and miR-34a identification, preclinical studies could be employed, like patient-derived organoids and xenograft models, to validate the functional impact of GAS5 modulation on YAP and miR-34a pathways. High-throughput transcriptomic and proteomic analyses can identify additional downstream effectors and potential biomarkers.

 After preclinical trials, these GAS5 modulations could be further validated by Phase I/II clinical trials. Based on robust preclinical data, early-phase clinical trials should be designed to assess the safety, tolerability, and preliminary efficacy of GAS5-targeted therapies like previously discussed antisense oligonucleotides in CRC patients. Stratification by YAP and miR-34a expression levels may help identify responsive subgroups.

 Finally, if it has been proven that GAS5 modulation could exert a significant effect, prospective cohort studies should evaluate circulating GAS5 levels, YAP and miR-34a status as prognostic or predictive biomarkers for CRC progression and therapeutic response. Addressing these research priorities systematically will ensure the field can accelerate the translation of GAS5 biology into tangible clinical benefits for CRC patients.

## Conclusion

 In conclusion, CRC is a very prevalent cancer around the world with the second-highest mortality despite advances in treatment. GAS5 LncRNA plays many roles in CRC progression with many different mechanistic pathways and interactions, affecting cell apoptosis, proliferation, invasion, migration, angiogenesis, and immune evasion, with various research concluding its downregulation contributes to cancer and drug resistance. GAS5 can modulate numerous miRNAs through its characteristic ceRNA function, as well as interact directly with proteins. The biomarker potential was also widely researched, as GAS5 can be detected in tissues, blood plasma, as well as endogenously, with more recent research using GAS5 as one of a few key components in a test for identifying CRC risk using blood. In therapeutic research, not many therapies are designed to target GAS5 directly as of writing this article, with limited studies on GAS5 interaction with natural products. There are no recorded clinical trials done on GAS5 therapeutics. In general, research on GAS5 has slowed down over the years, and more research is needed to better understand knowledge gaps on GAS5 mechanisms and interactions, and also potential therapeutic target studies. The potential of using GAS5 as a biomarker should be considered and tested in clinical trials, for further validation to realise this as a routine CRC screening test in laboratories. To realise its full potential in precision oncology, GAS5 must undergo multi-layered validation through integrative omics studies, functional genomics, and clinical biomarker pipelines. These efforts will clarify its context-specific roles and support its incorporation into diagnostic and therapeutic frameworks for CRC.

## Competing Interests

 The authors declare no conflict of interests.

## Ethical Approval

 Not applicable.
